# Nasal Lipopolysaccharide Challenge and Cytokine Measurement Reflects Innate Mucosal Immune Responsiveness

**DOI:** 10.1371/journal.pone.0135363

**Published:** 2015-09-14

**Authors:** Jaideep Dhariwal, Jeremy Kitson, Reema E. Jones, Grant Nicholson, Tanushree Tunstall, Ross P. Walton, Grace Francombe, Jane Gilbert, Andrew J. Tan, Robert Murdoch, Onn Min Kon, Peter J. Openshaw, Trevor T. Hansel

**Affiliations:** 1 Centre for Respiratory Infection (CRI), National Heart and Lung Institute (NHLI) at Imperial College, St. Mary’s Hospital: Mint Wing, Entrance C, Paddington, London, W2 1NY, United Kingdom; 2 GlaxoSmithKline, Stevenage, SG1 2NY, United Kingdom; 3 GlaxoSmithKline, Stockley Park West, Uxbridge, UB11 1BT, United Kingdom; Glaxo Smith Kline, DENMARK

## Abstract

**Background:**

**P**ractical methods of monitoring innate immune mucosal responsiveness are lacking. Lipopolysaccharide (LPS) is a component of the cell wall of Gram negative bacteria and a potent activator of Toll-like receptor (TLR)-4. To measure LPS responsiveness of the nasal mucosa, we administered LPS as a nasal spray and quantified chemokine and cytokine levels in mucosal lining fluid (MLF).

**Methods:**

We performed a 5-way cross-over, single blind, placebo-controlled study in 15 healthy non-atopic subjects (n = 14 *per protocol*). Doses of ultrapure LPS (1, 10, 30 or 100μg/100μl) or placebo were administered by a single nasal spray to each nostril. Using the recently developed method of nasosorption with synthetic adsorptive matrices (SAM), a series of samples were taken. A panel of seven cytokines/chemokines were measured by multiplex immunoassay in MLF. mRNA for intercellular cell adhesion molecule-1 (ICAM-1) was quantified from nasal epithelial curettage samples taken before and after challenge.

**Results:**

Topical nasal LPS was well tolerated, causing no symptoms and no visible changes to the nasal mucosa. LPS induced dose-related increases in MLF levels of IL-1β, IL-6, CXCL8 (IL-8) and CCL3 (MIP-1α) (AUC at 0.5 to 10h, compared to placebo, p<0.05 at 30 and 100μg LPS). At 100μg LPS, IL-10, IFN-α and TNF-α were also increased (p<0.05). Dose-related changes in mucosal ICAM-1 mRNA were also seen after challenge, and neutrophils appeared to peak in MLF at 8h. However, 2 subjects with high baseline cytokine levels showed prominent cytokine and chemokine responses to relatively low LPS doses (10μg and 30μg LPS).

**Conclusions:**

Topical nasal LPS causes dose-dependent increases in cytokines, chemokines, mRNA and cells. However, responsiveness can show unpredictable variations, possibly because baseline innate tone is affected by environmental factors. We believe that this new technique will have wide application in the study of the innate immune responses of the respiratory mucosa.

**Key Messages:**

Ultrapure LPS was used as innate immune stimulus in a human nasal challenge model, with serial sampling of nasal mucosal lining fluid (MLF) by nasosorption using a synthetic absorptive matrix (SAM), and nasal curettage of mucosal cells. A dose response could be demonstrated in terms of levels of IL-1β, IL-6, CXCL8 and CCL3 in MLF, as well as ICAM-1 mRNA in nasal curettage specimens, and levels of neutrophils in nasal lavage. Depending on higher baseline levels of inflammation, there were occasional magnified innate inflammatory responses to LPS.

**Trial Registration:**

Clinical Trials.gov NCT02284074

## Introduction

A range of inhaled and nasal challenges have been fundamental to our understanding of the pathophysiology of allergy and asthma [1] [2]. Challenge tests give a controlled stimulus after which serial measurements can be made, and the time course of responses can be assessed. Experimental challenge studies with components of microbes (including pathogen associated molecular patterns, PAMPs) and live viruses [3] give researchers the opportunity to study mucosal host-microbial interactions in terms of the specific and innate immune systems [4,5].

The respiratory mucosa of the nose has an intricate sentinel role interacting with bacteria, viruses and aeroallergens [6]. This is a complex and dynamic interaction that is mediated at a molecular level by PAMPs interacting with host pattern recognition receptors (PRRs) [7,8]. Lipopolysaccharide (LPS) is contained in the wall of Gram negative bacteria and in a range of microbes, and is a powerful PAMP with potent immunostimulatory properties through interactions with Toll-like receptor (TLR)4. LPS represents the prototypic PAMP involved in certain bacterial infections [7], atopy [9], and in acute lung injury [10]. Interestingly, TLR4 is involved in some viral infections [11]. House dust mite allergen has been shown to mimic an LPS-binding component of the TLR4 signalling complex and causes activation of airway structural cells [12,13]. Environmental exposure to LPS in childhood may facilitate development of tolerance to allergens [14], and this is one of a range of environmental microbial influences for the development of allergy and asthma [15].

LPS has been employed extensively as a challenge agent by David Peden and coworkers: with systemic administration by intravenous infusion [16], by inhalation from a nebulizer [17], by segmental bronchial challenge [18] and by nasal challenge [19–22]. In particular, the group of Lars-Olaf Cardell have carried out a series of studies with nasal LPS challenge: measuring CCL3 (MIP1α) in nasal lavage [23], evaluating a CXCR2 antagonist [24], demonstrating that allergen primes LPS responses [25], and showing that LPS challenge has augmented nasal cytokine release in allergic rhinitis [26].

Recently, synthetic absorptive matrices (SAM) have been used to sample mucosal lining fluid (MLF) in the nose, followed by measurement of mediators. Nasosorption has been employed in neonates [27], children with rhinitis [28] and in relation to nasal allergen challenge (NAC) [29]. The method of nasosorption has advantages in being non-invasive and being able to sample neat MLF, which is eluted in a known volume of buffer [30]. Because there is less dilution, nasosorption allows detection of higher levels of chemokines and cytokines than after nasal lavage. An additional non-invasive sampling method employs nasal epithelial curettage that has been employed to assess gene expression after nasal allergen challenge [31] and experimental rhinovirus infection [32].

In this study we carry out topical nasal ultra-pure LPS challenge in healthy non-atopic volunteers, and investigate the inflammatory response following a series of different doses of LPS. We employed serial nasosorption sampling and measured levels of soluble chemokines and cytokines using a multiplex immunoassay system. In addition, nasal epithelial curettage samples were taken for analysis of mRNA (transcriptomics) for intercellular cell adhesion molecule-1 (ICAM-1) that is elevated in the nasal mucosa in allergic rhinitis [33].

## Methods

### Ethics and Consent

This study was approved by the South West London Research Ethics Committee. Since this study was a topical challenge study for research purposes, and not for therapeutic benefit, the study did not require authorization from the UK Medicines and Healthcare Products Regulatory Agency (MHRA). The study was carried out on the Imperial Clinical Respiratory Research Unit (ICRRU) of St Mary’s Hospital in accordance with the Declaration of Helsinki and Good Clinical Practice (GCP) guidelines.

### Study Design

**Fig 1:**
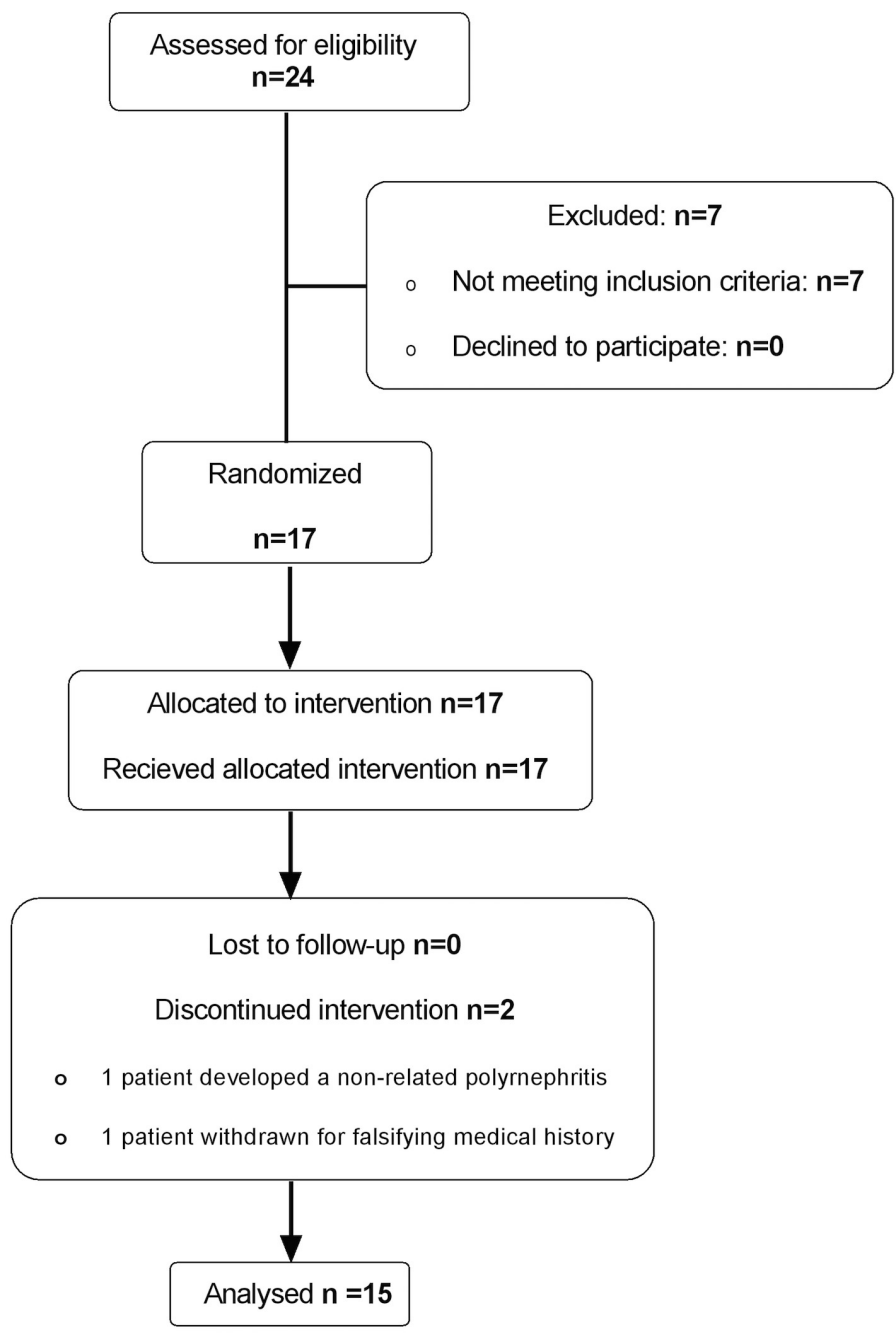
Consort Flow Chart.

**Fig 2:**
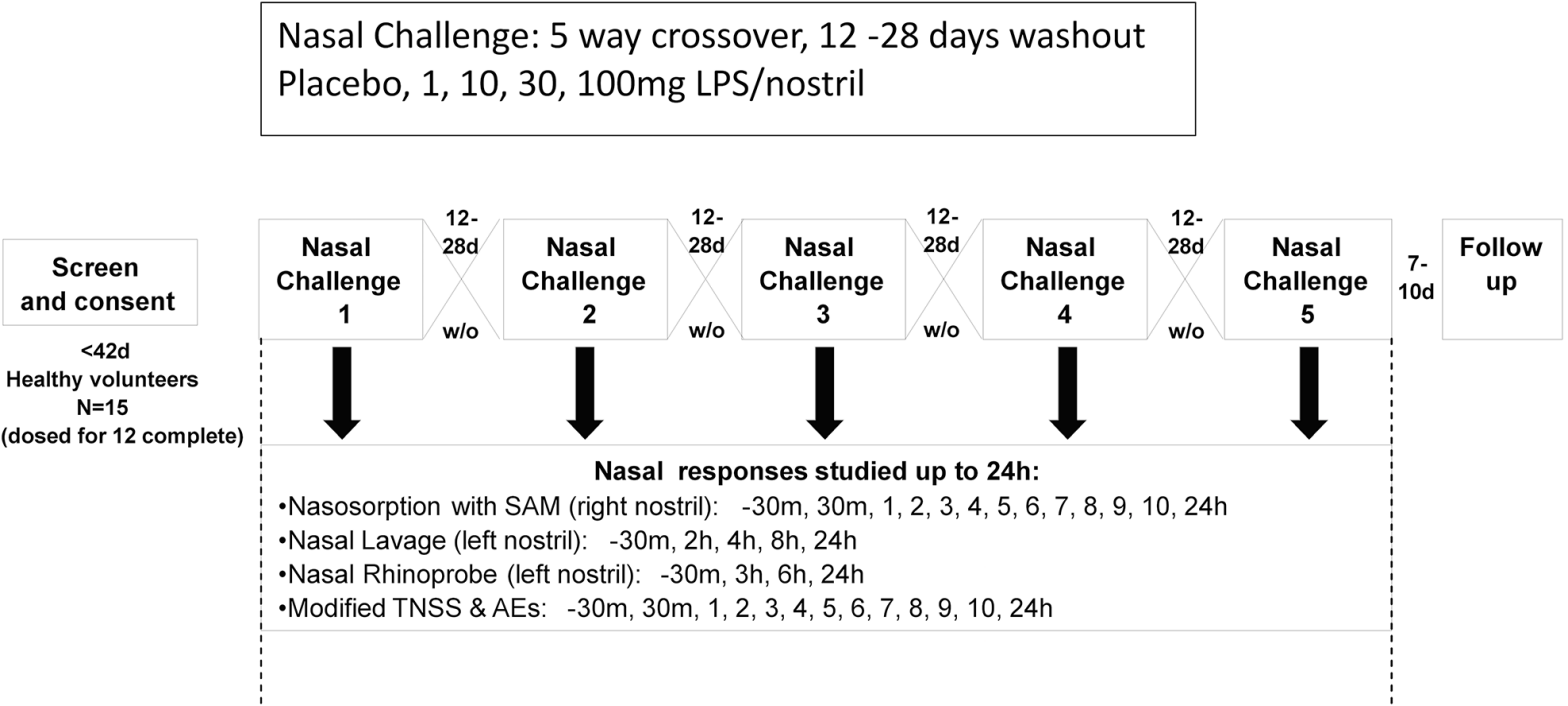
Nasal lipopolysaccharide challenge study design and procedures. 14 subjects completed the entire protocol (*per protocol*), while 1 subject only received nasal challenges at placebo and LPS. TNSS–total nasal symptom score. AE–adverse events

All healthy volunteers were recruited through newspaper advertising (**Fig 1**). The dose response to LPS was assessed by a 5-way crossover, randomized, single-blind, placebo-controlled study design (**Fig 2**). The dose randomization for the 5 arms (placebo, 1, 10, 30, 100μg LPS/nostril) was performed by an independent statistician, employing a balanced Latin square design to generate the order of challenges for each subject. The subjects were “blinded” to the dosage being employed. Subjects were assigned to a sequence in accordance with a randomisation schedule generated prior to the start of the study. The randomisation was balanced over the 5 nasal challenge sequences and was generated using RandAll, the GSK web-server based clinical trials randomisation system.

### Inclusion Criteria at Screening

Subjects were aged 18 to 60 years. They were current non-smokers for at least 6 months with a smoking history of <5 pack years. These individuals had no history of atopy, rhinitis, asthma, or other respiratory disease. Individuals with a history of malignancy or systemic disease were excluded, and use of any inhaled or oral anti-inflammatory medication was not permitted.

### LPS Administration

LPS solutions at concentrations of 10, 100, 300 and 1000μg/ml were prepared using ultra-pure LPS from *Escherichia coli* 0111:B4 (Invivogen, San Diego, CA).This is a high quality source of LPS, that is specific for TLR4 and does not contain bacterial lipoprotein contaminants that activate TLR2 (manufacturer’s specification). LPS potency was confirmed within our laboratory using the E-Toxate (*Limulus* Amoebocyte Lysate) kit (Sigma-Aldrich, Gillingham, UK). A standard curve and dilutions of the test LPS were prepared and mixed with the lysate. The ultra-pure LPS used in the clinical study contained 166–333 EU/μg, with the unit activity at the higher end of the manufacturer’s specification on the *Limulus* assay.

LPS and placebo were administered from a Pfeiffer Bidose nasal delivery device (Thermofisher, Epsom, UK) by a physician. A volume of 100μl was administered as a spray to each nostril, with a total dose per nostril of 1, 10, 30 or 100μg of LPS. Weights were recorded before and after each actuation from the Pfeiffer Bidose, to check that a 100μg weight of fluid had been delivered per nostril. A washout time of 12–28 days was employed between challenges.

### Symptom Scores

Modified total nasal symptom scores (TNSS) were recorded at the initial screening visit and before and at frequent intervals after nasal LPS challenge (-15 minutes, 30 minutes, then hourly from 1 to 10 hours and then at 24h post-LPS). Nasal congestion, rhinorrhea, sneezing, and nasal itch were scored from 0 to 3 (0: no, 1: mild, 2: moderate, 3: severe symptoms). The scores were then summed to give a final TNSS out of a maximum of 12.

We also measured safety parameters (temperature, heart rate, blood pressure and oxygen saturations), performed regular nasal mucosal inspections, and assessed peak nasal inspiratory flow (PNIF)

### Nasal Lavage

A modified nasal pool technique was adapted from the method of Greiff et al [34]. The subjects were seated in a forward-flexed neck position (60 degrees from the upright position) to prevent fluid from reaching the nasopharynx. To ensure adequate washing, the lavage fluid (5ml of 0.9% normal saline) was passed via a 10ml syringe slowly into the nasal cavity via an olive consisting of an oval, hollow, stainless steel device that was used to obstruct the nostril. The fluid was withdrawn into the syringe and gently flushed back into the nasal cavity 20 times over 1 minute.

Nasal lavage samples were initially centrifuged (4°C, 10 min at 400g), and the cell pellet resuspended in 0.01% dithiothreitol in phosphate buffered saline (PBS). The samples were gently agitated on a rolling mixer for 10 minutes and centrifuged; the supernatant was discarded; the pellet was resuspended in PBS at 1 to 2 million cells/mL, and cytospin slides were prepared. Differential leukocyte counts were determined by assessment of 400 leukocytes on the stained cytospin.

### Nasosorption

Nasosorption was performed with strips of synthetic absorptive matrix (SAM; Fibrous Polyester; Hunt Developments, Midhurst, West Sussex, UK). The SAM was inserted under direct vision into the nasal cavity, the length being applied laterally against the anterior inferior turbinate. Nose clips were used to ensure good contact with the mucosal surface. Pre-chilled assay buffer (PBS, bovine serum albumin (BSA) 1%; Tween-20 0.05%, sodium azide 0.08% (Milliplex Assay Buffer, cat no. L-AB, Merck Millipore, Billerica, Mass., USA), was dispensed in 500μL aliquots into filter cups within Eppendorf tubes (Costar spin-X, cellulose acetate). After removal of the SAM strips from the nose, they were placed in the assay buffer, and spin filtration was performed (4°C for 5 min at 16,000 g). The eluate was collected and stored as aliquots at -80°C, prior to assay of chemokines and cytokines by a multiplex immunoassay (Mesoscale Diagnostics, MSD, Gaithersburg, Md, USA).

### Cytokine levels in Nasosorption Samples

Two custom 7-spot MSD plates (MSD) were used for measuring the concentrations of CCL3 (MIP-1α), IL-1β, IL-6, CXCL8 (IL-8), TNF-α, IFN-α, IL-10 (Plate 1) and IFN-γ, GM-CSF, CCL2 (MCP-1), CXCL10 (IP-10), IL-12p70, IL-17 (Plate 2). The plates provided are pre-coated with antibody for the specific analytes. Diluent 2 (MSD) (25μl) was added to each well and the plate was incubated for 30 min while being vigorously shaken. All incubations in the MSD immunoassay were done on an orbital shaker at 1000 rpm at room temperature. 25μL of the sample or calibrator was added to each well. A standard curve of calibrators (10000, 2500, 625, 156.3, 39.1, 9.8 and 2.4pg/mL) was run in duplicate. Controls of known concentrations (625pg/mL and 9.8pg/mL) were done in quadruplicate. Three replicates of each sample were added. After an incubation of 2 hours on a shaker with the sample/calibrator the plates were washed three times with PBS + 0.05% Tween and then 25μL of 1X Detection Antibody Solution was added to each well. After incubation for 2 hours with vigorous shaking the plate was washed 3 times with PBS + 0.05% Tween. 150μL of 2X Read Buffer was added to each well and the plate was read immediately using a MSD Sector Imager 6000. The raw data was analysed using MSD Discovery Workbench 3.0 software. The concentration of each cytokine was calculated from the standard curve of the Calibrators.

### Nasal Epithelial Curettage

A single nasal curettage was performed at baseline and again at 3, 6, and 24hrs to obtain epithelial cells for mRNA gene expression studies using a plastic nasal curette (Rhinoprobe, Arlington Scientific). All nasal scrapes were performed after the collection of nasosorption and nasal lavage samples. Nasal scrapes were taken from the posterior aspect of the inferior turbinate, without local anaesthesia, collecting a single 5mm mucosal sample. Nasal scrapes were placed in labelled sterile tubes containing TRIzol reagent (500μl) and stored at -80°C.

### Analysis of ICAM-1 mRNA expression from nasal Rhinoprobe samples by RT-qPCR

Nasal scrapes samples were placed in TRIzol Reagent (Invitrogen, Carlsbad, CA, USA) and stored at -80°C until the total RNA was purified. Total RNA was extracted using RNeasy Mini-kit (Cat. No. 74104, Qiagen Gmbh, Hilden, Germany) and treated with RNase-free DNase (Cat.No. 79254, Qiagen) according to the instructions of the manufacturer. RNA concentrations were determined with a NanoDrop 8000 Spectrophotometer (Thermo Scientific, Wilmington, DE, USA). cDNA was synthesized using the Applied Biosystems High Capacity cDNA Reverse Transcriptase Kit (catalogue number 4368813) with 3ng of RNA in a final reaction volume of 20μl. The reaction was incubated at 25°C for 10 minutes, followed by 2 hours at 37°C. qPCR was performed using the Taqman universal PCR master mix (Applied Biosystems Catalogue number 4318157) on an 7900HT Fast Real Time PCR System. Six replicates were run per sample. Sequences of Taqman primer/probes were as follows: Human ICAM-1 forward primer 5’-GCTCTGCAACCCTGGAGGT-3’; reverse primer 5’-GGCCATACAGGACACGAAGC-3’; Probe CCAGCTTATACACAAGAACCAGACCCGG. All data was normalised using Human GAPDH as a housekeeper gene. GAPDH forward primer 5’-CAAGGTCATCCATGACAACTTTG-3’; Reverse primer 5’-GGGCCATCCACAGTCTTCT-3’; Probe ACCACAGTCCATGCCATCACTGCCA.

### Statistical analysis

Biomarker data was available for 15 subjects although subject number 3102 withdrew after 2 periods. For all biomarkers, the lower limit of quantitation (LLOQ) was taken as 9.8pg/ml and a value of LLOQ/2 (= 4.9pg/ml) was used for all values less than the LLOQ. The median was taken of the 3 adjusted readings and used in the analysis. The area under curve (AUC) from 0-10h of the observed data were calculated for each dose of LPS, and responses after LPS challenge described as changes relative to placebo AUC 0-10h. The distribution of the AUC data was tested using a Shapiro-Wilks test and significant deviations from normal data distribution were seen. Therefore non-parametric statistics (Wilcoxon signed rank test) were applied throughout.

Statistical analysis of ICAM-1 mRNA data was performed in SAS v9.2 using a mixed model analysis of variance. All subjects received each of the treatments at a separate visit and within each visit a response across time was obtained. Due to the complex design of the study ‘time’ was included in the model as a repeated measures and ‘subject/ visit’ was included as a random effect to allow the appropriate error structure to be used for assessing LPS effects at each time point. The response used was logged gene abundance and log GAPDH (glyceraldehyde-3-phosphate dehydrogenase) was included as a covariate so that the resulting estimates of the mean fold changes from placebo were normalised.

## Results

### Baseline Demographics

Subjects were recruited through newspaper adverts and all visits took place between November and April (**Fig 2**). Following initial entry of 17 subjects, a total of 14 subjects completed all study visits and sampling procedures (*per protocol* group). A single individual received 2 doses (placebo and LPS at 100μg) and was included in the analysis, so that 15 subjects were studied. Subjects ranged in age from 21 to 57 years (mean 38 years), and there were 10 males and 5 females.

One individual withdrew from the study following development of an infective pyelonephritis 10 days after a low dose of LPS (1μg), this being the first nasal challenge, and the event was considered to be “not clinically related to LPS administration”. A second individual was withdrawn from the study after 2 of the 5 planned nasal challenges, as they had withheld medical information that excluded their participation. These 2 subjects were excluded from the analysis.

### Clinical Symptom Scores

None of the participants experienced any significant clinical symptoms during the course of their study participation. Following nasal LPS administration there were no visible signs of inflammation on direct examination of the nasal mucosa by a physician.

### Levels of Cytokines and Chemokines in Nasal MLF

**Fig 3:**
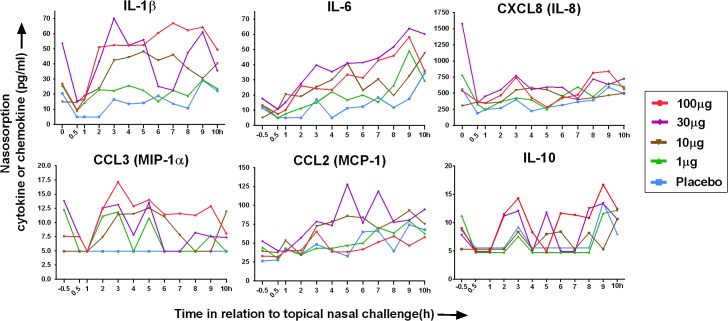
Median levels of chemokines and cytokines (pg/ml) in nasal mucosal lining fluid (MLF) are shown after nasal spray challenge with 4 different doses of LPS and a placebo (see key below). Lower limits of detection (LLOD) for cytokines and chemokines were as follows: IL-1β (5.0pg/ml), IL-6 (5.0pg/ml), CXCL8/IL-8 (10.0pg/ml), CCL3/MIP-1α (5.0pg/ml), CCL2/MCP-1 (25.0pg/ml), IL-10 (5.0pg/ml). Time -0.5h refers to nasosorption performed 30 min prior to topical nasal challenge, and this varies considerably between individuals due to variations in individual’s microbial flora and mucosal immune responses. Nasal lavage was performed after nasosorption at -0.5h, but prior to nasal challenge, in order to partially wash the nose free of baseline inflammatory microbes and mediators, but causes detectable levels of inflammatory mediators at 0.5h to decrease markedly from -0.5h. There is a tendency for nasosorption levels of mediators to gradually increase from 0.5h to 10h in the placebo arm, but levels of inflammatory cytokines and chemokines after LPS are generally higher.

Following nasal LPS challenge there was a dose response for IL-1β, IL-6, CXCL8, CCL3 and IL-10 levels in nasal SAM eluate. Median levels of these cytokines and chemokines over time are shown in **Fig 3**. Although there was an increase in the response to nasal LPS at doses up to 30μg, the response at 30 and 100μg was approximately equivalent.

**Fig 4:**
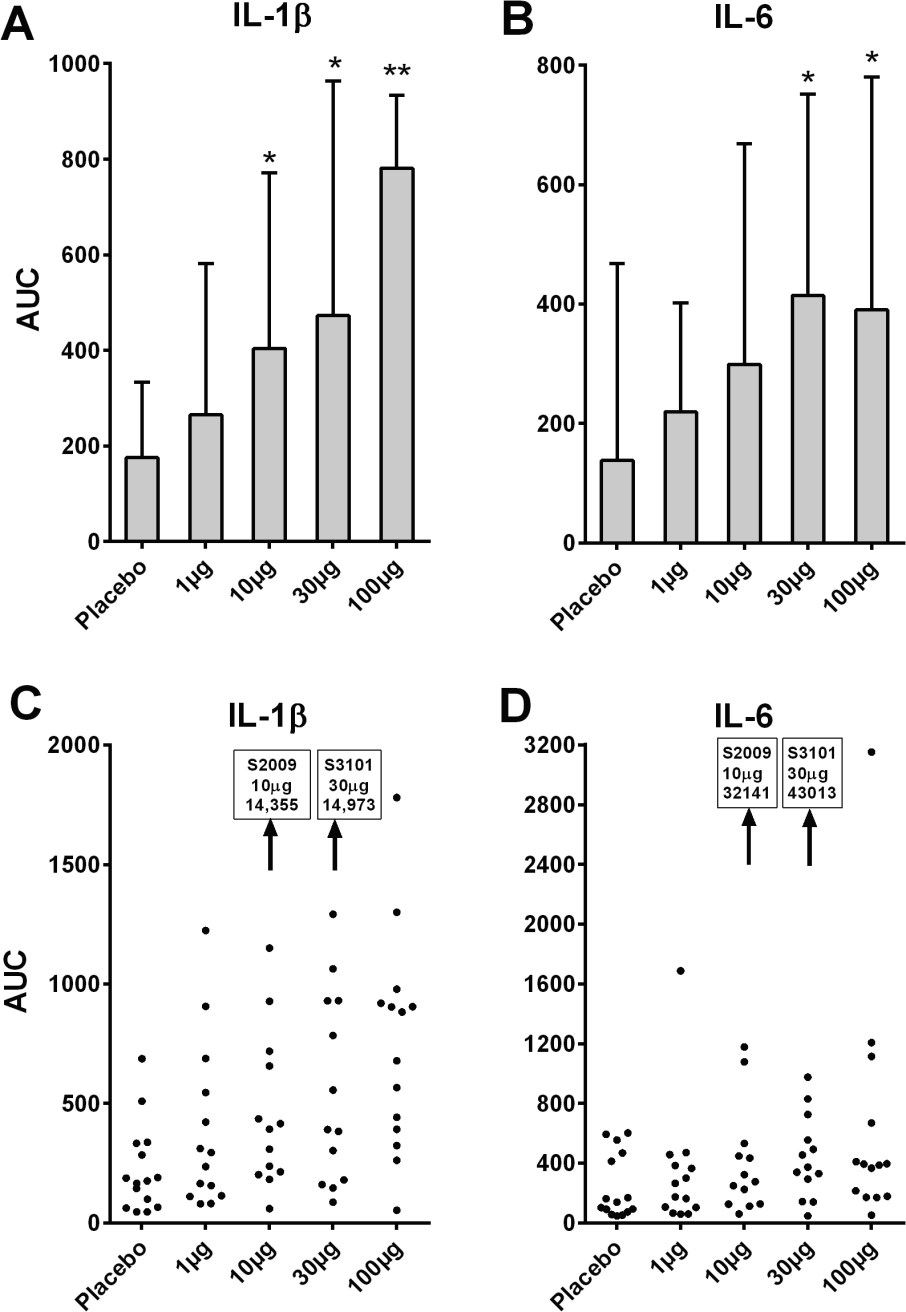
Levels of interleukins (IL) in nasosorption eluates presented as area under the curve (AUC) for times 0.5-10h in relation to topical nasal challenge (n = 14 or 15). AUC is derived from from 0.5 since the baseline sample at -0.5h is pre-nasal lavage and is highly variable due to difference in individuals in terms of nasal microbes and inflammatory mediators. A) IL-1β as medians with quartiles; B) IL-6 as medians with quartiles; C) IL-1β AUC data for individual subjects; D) IL- AUC data for individual subjects. Note the idiosyncratic large responses of subject 2009 after nasal challenge with 10μg LPS, and subject 3101 after nasal challenge with 30μg LPS, these responses being too large to be presented on the y axis.

The Area Under the Curve (AUC) for individual biomarkers measured at 0.5 to 10h was compared with placebo. Following LPS there were significant increases in AUC for IL-1β, IL-6, CXCL8, and CCL3 (**Table 1**). IL-10, IFN-α and TNF-α were increased significantly at 100μg alone. There were particularly marked IL-1β and IL-6 responses and AUC data is shown in response to nasal LPS dose as medians with quartiles, and individual AUC responses in **Fig 4**.

**Table 1:** Statistical analysis of levels of cytokines and chemokines in nasal mucosal lining fluid^1^.

Cytokine or chemokine	Nasal challenge LPS, 1μg	Nasal challenge LPS, 10μg	Nasal challenge LPS, 30μg	Nasal challenge LPS, 100μg
IL-1β	ns	<0.05	<0.05	<0.01
IL-6	ns	ns	<0.05	<0.05
CXCL8 (IL-8)	ns	ns	<0.05	<0.01
CCL3 (MIP-1α)	<0.05	ns	<0.05	<0.001
CCL2 (MCP-1)	ns	<0.05	ns	ns
IL-10	ns	ns	ns	<0.05
IFN-α	ns	ns	ns	<0.05
TNF-α	ns	ns	ns	<0.05

^**1**^Footnote: Area Under the Curve (AUC) for individual cytokines or chemokines measured at 0.5 to 10h was compared with placebo. The non-parametric Wilcoxon signed rank test was used for statistical analysis. Probability (p) values are presented, ns = non significant. The levels of the following cytokines and chemokines were not significantly altered by LPS: IFN-γ, GM-CSF, CXCL10 (IP-10), IL-12p70, IL-17.

### Idiosyncratic Responses (2)

Examining the levels of cytokines and chemokines in nasal SAM eluates from individual subjects, it was noted that 2 subjects had unexpectedly large responses to LPS on a single occasion during dose-escalation. This is illustrated in **Fig 4**(lower portion) that shows levels of cytokines and chemokines as AUC from 0.5h to 10.0h. These markedly elevated responses occurred in subject 2009 following 10μg LPS, and in subject 2101 following 30μg LPS. With regards to the order of challenges, subject 2009 received 10μg as their second dose (100μg, 10μg, 1μg, placebo, 30μg), while subject 3101 received 30μg LPS as their first dose (30μg, 100μg, 1μg, placebo, 10μg). This suggests that sensitisation to LPS is not the explanation for these large idiosyncratic responses.

**Fig 5:**
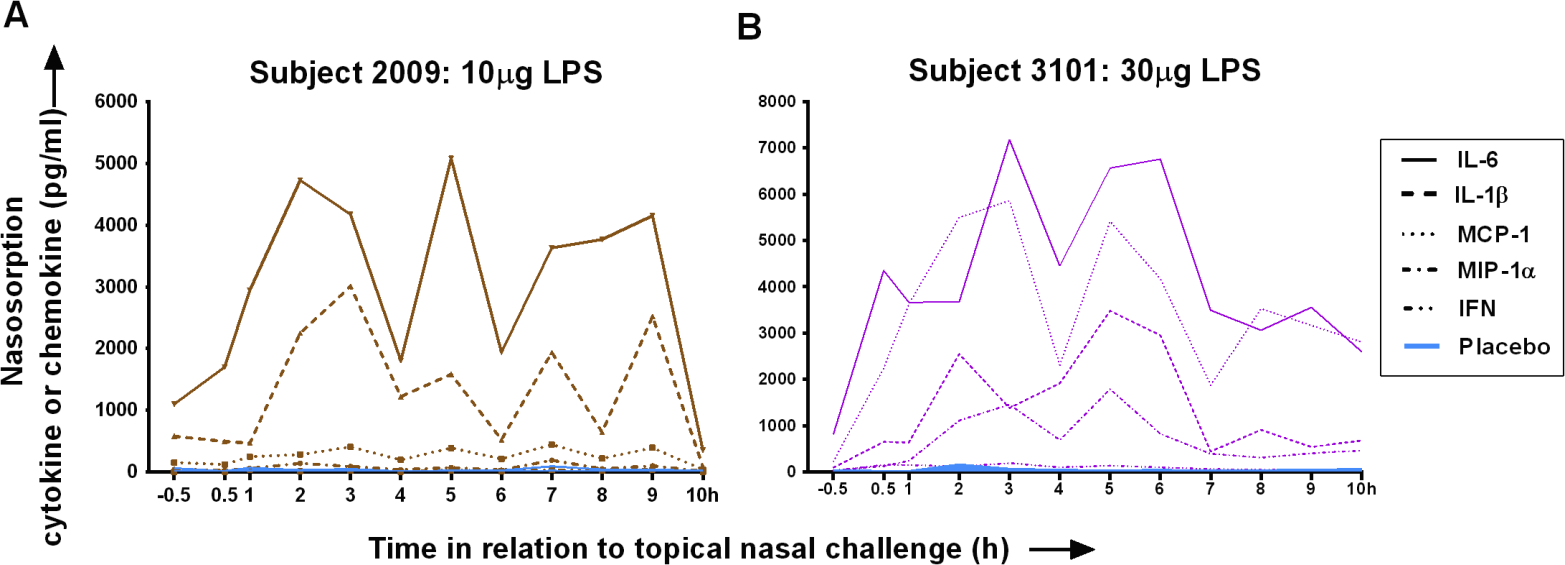
Idiosyncratic large nasal responses to lipopolysaccharide (LPS) on 2 occasions. Raw data for levels of chemokines and cytokines (pg/ml) in nasal mucosal lining fluid (MLF). Data is presented for individual subjects: A. Subject 2009 after nasal challenge with 10μg LPS. B. Subject 3101 after nasal challenge with 30μg LPS. Note that baseline levels of cytokines and chemokines at -0.5h are markedly raised, and come down to only a small extent after nasal lavage at 0.5h. It is suggested that these individuals may have had a non-symptomatic alteration in their nasal microbial flora prior to LPS challenge on these opccasions: possibly the individuals had a subclinical viral infection or bacterial colonisation.

Following the 2 idiosyncratic responses to nasal LPS, the raw data for nasal SAM eluate levels of cytokines and chemokines is presented in **Fig 5**. In subject 2009 the response was mainly in terms of IL-6 and IL-1β, while in subject 3101 CCL2 (MCP-1) was also elevated. It was noteworthy that on the occasion of both these idiosyncratic responses, there were high baseline levels of nasal MLF IL-1β and IL-6.

### Levels of Nasal Lavage Neutrophils

**Fig 6:**
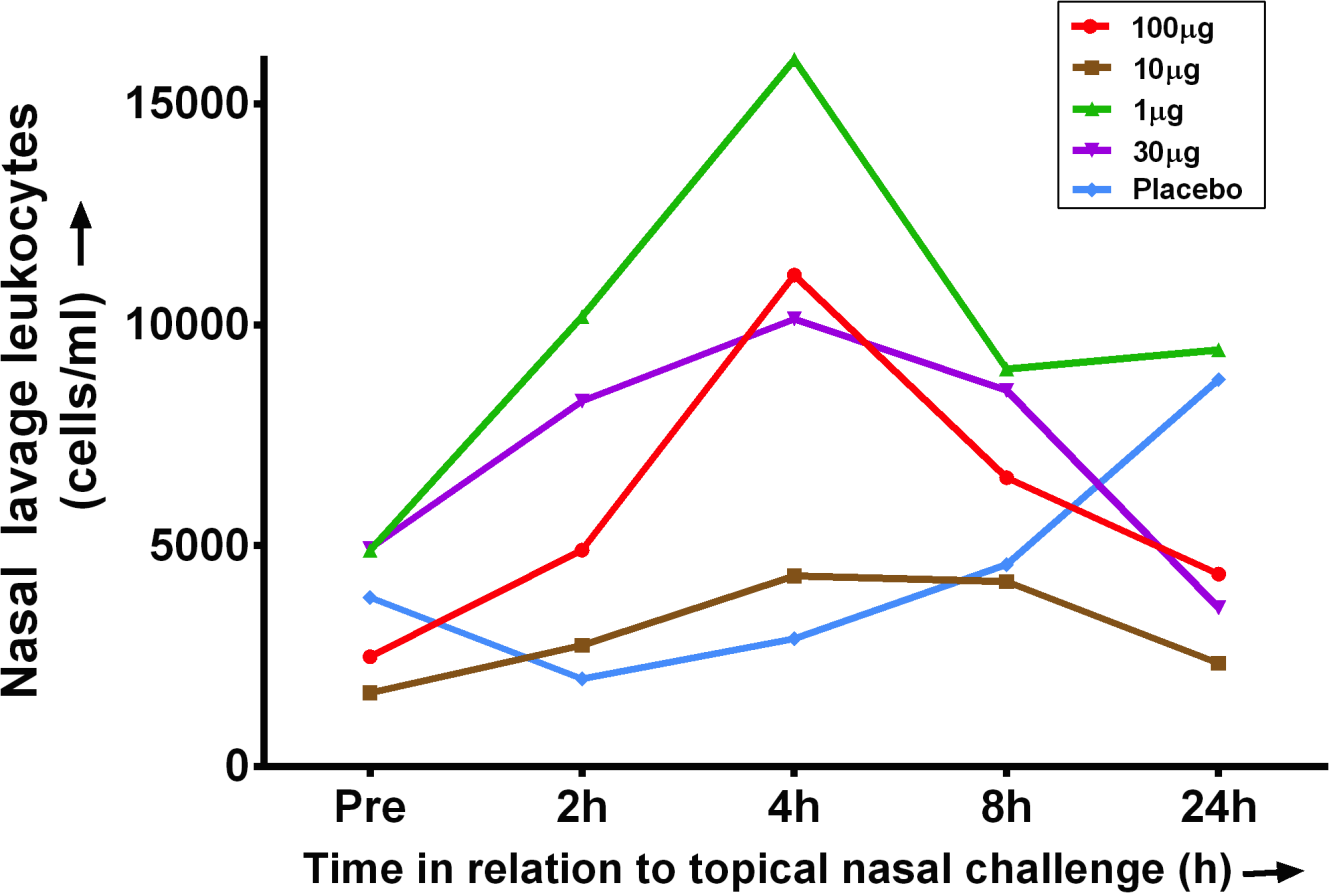
Nasal lavage leukocyte counts (medians expressed as cells/ml) following nasal LPS challenge. Cells were counted in a modified Neubauer chamber, and a leukocyte differential performed on a stained cytospin observed under a light microscope.

There was an increase in the number of neutrophils in nasal lavage from baseline at 4h with doses of LPS at 1, 30 and 100μg (**Fig 6**). However, changes did not reach significance, and there was not dose-related. This is in keeping with our experience that nasal lavage is a somewhat unreliable sampling method, and that subsequent cell counts can be variable.

### ICAM-I mRNA

**Fig 7:**
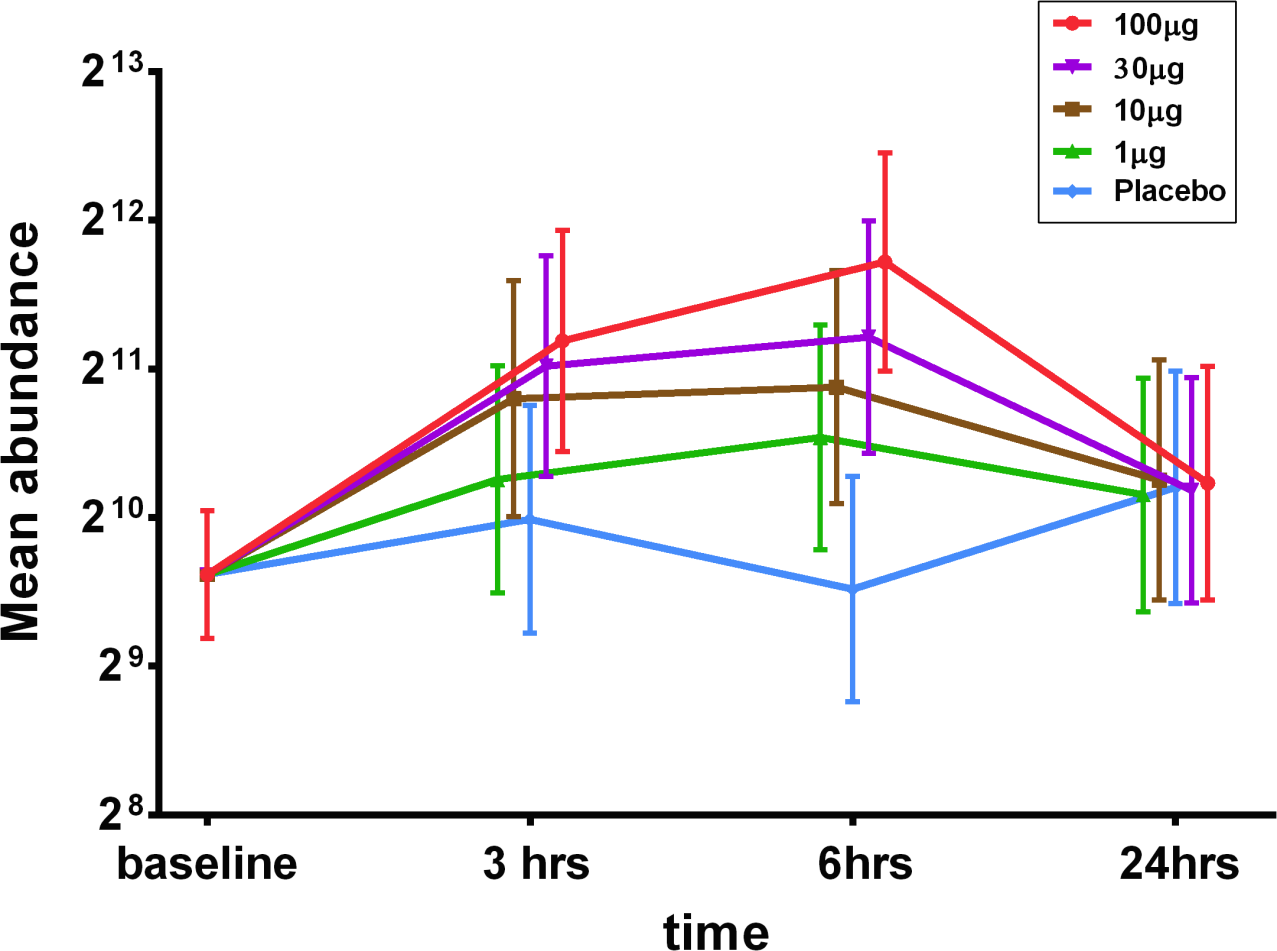
ICAM-1 mRNA abundance within nasal curettage samples after nasal LPS challenge, Data is shown as means with 95% confidence intervals. Relative abundance of expression with Reverse Transcriptase quantitative Polymerase Chain Reaction (RT-qPCR) using normalized data and (glyceraldehyde-3-phosphate dehydrogenase) (GAPDH) as a housekeeper gene (see Methods).

ICAM mRNA expression was measured in nasal curettage samples by quantitative PCR (qPCR). Samples were taken 30 minutes before LPS challenge and at 3, 6 and 24 hours post challenge. A dose dependent increase in ICAM-1 mRNA expression was observed following LPS treatment (**Fig 7**). The maximal increase in ICAM-1 mRNA over placebo was observed at 6 hours post LPS challenge: 1μg LPS (p<0.05), 10μg LPS (p<0.01), 30μg LPS (p<0.01) and 100μg LPS (p<0.0001). A significant increase over placebo was also observed at 3 hours for the 30μg and 100μg groups (both at p<0.05). ICAM-1 mRNA expression levels returned to baseline at 24h post LPS treatment.

## Discussion

This 5-way crossover study documents the nasal mucosal immune response to LPS challenge by performing serial non-invasive sampling (nasosorption and nasal epithelial curettage) and then measuring levels of cytokines and chemokines together with mRNA for ICAM-1. Original features of this report include the use of ultrapure LPS, serial nasal sampling by the novel method of nasosorption, employing LPS at a range of 4 doses, measuring a range of cytokines and chemokines using a multiplex immunoassay, and assessment of mRNA in epithelial curettage specimens. We were able to observe a dose response to LPS up to 30μg in terms of significantly increased levels of IL-1β, IL-6, CXCL8 and CCL3, which suggests that the dose of 50μg per nostril employed by Cardell and co-workers is optimal in non-atopic subjects [23] [26,35]. Other nasal LPS challenge studies have demonstrated increases in IL-6 [20,36], CXCL8 [21,35] and CCL3 [23].

In the situation of a nasal topical challenge and wishing to take a series of repeated samples from the nasal mucosa, nasosorption of mucosal lining fluid has advantages over nasal lavage. In a study if repeated sampling in healthy volunteers, it was found that nasal lavage levels of a range of mediators were approximately 10-times lower than those from nasosorption eluates [37]. This was confirmed in a study of levels of IL-4, IL-5 and IL-13 after nasal allergen challenge (NAC), where levels of these interleukins were higher in nasosorption eluates, and frequently undetectable in nasal lavages [38]. In a recent study it was found that repeating nasal lavage within 0.5 to 4h caused markedly decreased levels of mediators, and they concluded that nasal lavage can only be performed once daily to get comparable results [39].

Using a modified nasal lavage collection method, a recent nasal LPS challenge study failed to show significant cytokine responses in allergic rhinitis patients, but amplified responses occurred when nasal allergen challenge preceded nasal LPS challenge [26]. The reason our study was able to document a dose response was probably because we utilized nasosorption to obtain nasal MLF, while other studies have generally measured more dilute nasal secretions present in nasal lavage.

An interesting finding in the study was that in 2 subjects there were large idiosyncratic responses to submaximal doses of LPS. Whereas the median response following higher doses of LPS may be up to 5 times greater than that after placebo, the idiosyncratic reactions involved IL-6 and IL-1β responses of greater than 1000 times those of placebo. Despite these large inflammatory reactions, subjects reported no symptoms, and there were no visible signs of nasal inflammation. When we assessed the sequence of LPS administrations, there was no evidence of a sensitization or tolerization process occurring with earlier doses. The phenomenon of tolerance to endotoxin has been demonstrated when low dose endotoxin is administered nasally up to 3 times on consecutive days [19,22],

The idiosyncratic exaggerated reactions occurred in one subject after 10μg LPS and in another subject at 30μg LPS. The upper respiratory tract is now recognised to contain a vast array of commensal and pathogenic bacteria included within the nasal microbiome, and these may interact with aeroallergens [40] [9]. In asymptomatic healthy individuals there is frequently carriage of the potential pathogens *Staphylococcus aureus* and *Streptococcus pneumoniae*. There is then extensive crosstalk among PAMPs, TLRs and other innate PRRs in the mucosa [7]. Interestingly the mucosal relationship with bacteria and viruses is altered in the asthmatic or allergic airway. In particular, TLR4 signalling in stromal cells has been shown in mice to be critical for initiation of allergic Th2 responses [41].

We are not currently able to explain these idiosyncratic responses, but each case there were high levels of IL-1β and IL-6 in nasal mucosal lining fluid before challenge. This leads us to speculate that the idiosyncratic reactions relate to pre-activation by variations in nasal exposure to aeroallergens, viruses or bacteria.

In addition LPS caused a dose response in terms of induction of gene expression for ICAM-1, that is upregulated following inflammation, and acts as an epithelial receptor for human rhinoviruses (HRV). ICAM-1 is upregulated in the nasal mucosa of allergic rhinitis patients [33], and HRV causes activation of epithelial cells through this receptor. Measurement of soluble-ICAM-1 in nasal MLF would confirm whether the increased ICAM-1 gene expression relates to increased production of protein.

In conclusion, this study demonstrates a dose response to nasal LPS challenge in terms of levels of cytokines and chemokines measured in serial nasal MLF samples, and also with raised levels of ICAM-1 mRNA being found in serial nasal epithelial curettage samples. However, we also highlight the occasional phenomenon of an idiosyncratic large response to LPS, which is not dose-related, and that occurred in 2 subjects on single occasions.

In future studies this method, we intent do examine mucosal immune responses to a series of different challenge agents ranging from allergens, diverse microbial components and live viruses. We will also study the longitudinal responses to LPS and other agents over time and to document the influences of one nasal challenge agent on another (as has been done for nasal allergen challenge followed by LPS challenge [26]). Finally, in assessing the response to these nasal challenges in patients with allergic rhinitis and asthma, it may be possible to characterize mucosal immunology in individual patients as a basis for rational therapy.

## Supporting Information

S1 CONSORT Checklist CONSORT Checklist. (DOC)

S1 File Data Files. (XLS)

S1 Protocol Study Protocol. (DOC)
