# Supplementary material for: Nasal Lipopolysaccharide Challenge and Cytokine Measurement Reflects Innate Mucosal Immune Responsiveness
Source: PLoS One. 2015 Sep 14;10(9):e0135363. doi: 10.1371/journal.pone.0135363 (PMC4569396; doi:10.1371/journal.pone.0135363)
Supplement: S1 Protocol — (DOC) [file pone.0135363.s003.doc]

**Study Title**

**Nasal lipopolysaccharide (LPS) challenge in healthy volunteers (HVs):**

**investigation of tolerability, dose response and the expression profile of intercellular adhesion molecule-1 (ICAM-1)**

| Study Protocol Number | ICRRU/2010/001 |
| --- | --- |
| Version No and Date | Version 01, dated 12^th^ May 2010 |
| Principal Investigator | Dr. Trevor Hansel |
| Co-Investigator | Prof. Peter Openshaw |
| ICRRU Staff | Andrew Tan (Clinical Research Manager)  Linda Green (Senior Research Nurse)  Julie Gent (Research Nurse)  Grant Nicholson (Research Scientist) |
| Study Centre | Imperial Clinical Respiratory Research Unit (ICRRU)  St. Mary’s Hospital  Mint Wing, First Floor  Paddington  London W2 1NY |
| Sponsor | Imperial College |
| Funder | GlaxoSmithKline in a Collaborative Clinical Research Study |
| GSK Staff | Dr. Robert Murdoch (GSK Study Responsibility)  Dr. Jeremy Kitson (Research Scientist)  Ms. Jane Gilbert (Statistics) |

**Study Title: Nasal lipopolysaccharide (LPS) challenge in healthy volunteers (HVs): investigation of tolerability, dose response and the expression profile of intercellular adhesion molecule-1 (ICAM-1)**

1. **Introduction**

Chronic Obstructive Pulmonary Disease (COPD) and severe asthma are neutrophilic inflammatory diseases of the airways for which there is the need to identify novel effective anti-inflammatory therapy ^(1;2)^. Nasal and inhaled allergen challenge are established models for the clinical assessment of anti-inflammatory agents for allergic rhinitis (AR) and asthma, especially for agents that target Th2 cells, mast cell degranulation and eosinophil recruitment. There is a need for a related phase II clinical pharmacology challenge model for testing anti-inflammatory drugs that target neutrophil and monocyte influx, and act on endothelial and epithelial activation ^(3)^. In this proposal we hope to establish the reproducibility and dose response of ultrapure lipopolysaccharide (LPS) (Invivogen) delivered by nasal spray to healthy volunteers. LPS is found in the wall of Gram-negative bacteria and contains pathogen-associated molecular patterns (PAMPs) that act on host pattern recognition receptors (PRRs), including CD14 and toll-like receptor-4 (TLR4 agonist) ^(4;5)^.

Infection with human rhinovirus is a major cause of asthma exacerbations and is thought to be a significant contributor in COPD and other respiratory diseases. It is estimated that approximately 90% of rhinovirus serotypes use intercellular adhesion molecule-1 (ICAM-1) as their receptor. Induction of ICAM-1 expression promotes recruitment and activation of inflammatory cells. Treatments impacting on ICAM expression may offer a novel target to reduce the frequency and severity of upper and lower respiratory tract infection and hence reduce exacerbation rate in high risk patients. Nasal LPS challenge offers a model to upregulate ICAM expression and subsequently may have utility in early drug development studies determining “Proof of Mechanism”.

It has long been recognised that there is “one airway”, with a strong functional and immunological relationship between the nose and bronchi ^(6;7)^, especially in terms of infiltrating leukocytes and inflammatory mediators when comparing allergic rhinitis and allergic asthma ^(8)^. There is also the insight that patients with respiratory disease commonly have inflammation of both the airways and nasal passages, with a similar type of inflammatory infiltrate in lower and upper airways.

It is possible to obtain repeated samples of nasal exudates and mucosa cells before and after nasal challenge in a relatively non-invasive way by techniques such as nasal filter paper ^(9)^ ^(10)^, nasal lavage ^(11)^., and nasal epithelial scrapes (Rhinoprobe curette) ^(12)^.

- Synthetic absorption matrix (SAM) strips can be placed on the turbinates to absorb nasal lining fluid ^(13)^. The nasosorption method has the advantage of directly sampling nasal secretions which are less diluted, and releasing fluid on spin filtration, with less protein binding than with filter paper. The SAM method of sampling can therefore pick up protein signals which are below the detection limits of nasal lavage.
- Nasal lavage is commonly performed to isolate leukocytes from the nasal mucosal surface by the nasal pool method of Greiff and colleagues ^(11)^.
- A plastic nasal curette (Rhinoprobe, Arlington Scientific) can be used to obtain epithelial cells for cytology ^(14)^ and mRNA gene expression studies ^(15;16)^. This method of sampling is almost painless, and has been extensively employed in adults and infants ^(17)^.

Nasal allergen challenge (NAC) is a model that has been increasingly used instead of inhaled allergen challenge to assess both topical and systemic new therapies for asthma and allergy ^(18)^. Using the NAC in phase II of drug development for asthma and allergic rhinitis it is possible to provide an early and reliable “Go/No Go” decision before embarking on large-scale, long-term and costly phase III studies.

Using our experience with NAC and novel sampling methodologies, we would like to develop a model of nasal challenge with LPS. Bacterial LPS is an active component of cigarette smoke ^(19)^, and nasal challenge with LPS has been studied in a variety of different models causing release of mediators such as TNF-α and MIP-1α ^(20-26)^. These mediators have been detected in nasal lavage, and we expect nasosorption to achieve much higher detectable levels of mediators.

**Previous administration of LPS to Man**

a. There is considerable published experience with administration of LPS to man.

Systemic injection of LPS intravenously causes a mild fever and decrease in FEV_1_ in healthy volunteers at 2ng/kg ^(27;28)^(12383} and at 4ng/kg ^(29)^.

Inhaled LPS has been administered from a nebulizer at doses of 50μg ^(30;31)^ and 60μg ^(32)^.

Nasal LPS has been used in man with no reports of any local or systemic symptoms or adverse effects being caused ^(20-26)^. In subjects given a nasal spray, LPS at 25μg per nostril has been given in two studies ^(24;25)^ and LPS at 50μg per nostril was employed in a further study ^(26)^. The LPS source used in these studies is Sigma (catalogue no. L3129, E. coli serotype 0127:B8).

b. In mice and rats, acute LPS nasal challenge is routinely performed with 2.5mg/kg, and is given as 50μg LPS as an acute intratracheal dose. The source of LPS used in these studies is Sigma (catalogue no. L3129, E. coli serotype 0127:B8).

c. We propose a highest dose of LPS of 100μg per nostril (InvitroGen Ultrapure) since Sigma LPS is 1.5 to 3 times more potent. Hence, 100μg of InvitroGen LPS has a potency similar to that of 50μg of Sigma LPS, and this dose of Sigma material has been well tolerated in man ^(26)^.

# 2. Study Hypotheses and Objectives

The purpose of this study is to develop a safe and effective challenge model to LPS in terms of induction of ICAM-1, and so provide a robust framework for the clinical assessment of future anti-inflammatory therapies directed specifically against ICAM-1.

- 1. **Hypothesis**

The nasal ICAM-1, cellular and cytokine/chemokine responses will be reproducible and show a dose response in relation to the amount of LPS used to challenge.

**2.2 Primary Objective**

The principal research questions (primary end point, PEP):

- Can we develop a well tolerated, robust and reproducible (validated) nasal LPS challenge system in terms of ICAM-1 induction ?
- Can we document a dose response and time course to nasal LPS in terms of cell influx, cytokine and chemokine release, and nasal epithelial curettage (Rhinoprobe) mRNA expression ?
- To assess the unstimulated expression of ICAM-1 in nasal epithelial scrapes.
- To provide a nasal challenge method with LPS to enable a “proof of target pharmacology” for a therapy directed against ICAM-1 induction. This may also permit dosage optimisation in relation to clinical efficacy studies.

1. **Study Design**

5-way crossover study, randomized, single-blind, placebo-controlled, dose-response to LPS.

A single cohort of 15 healthy volunteers will be studied in a 5-way cross-over study (4 doses of LPS versus placebo), for a minimum of 12 to complete.

A balanced Latin square randomized design will be used.

Total study duration: 6-9 months

1. **Study population**

For this clinical study it is proposed to study healthy volunteers with no history of allergy, nor any other significant illnesses or therapy.

15 healthy volunteers will be recruited, for a minimum of 12 to complete

**4.1 Inclusion criteria**

- Males and females aged 18 to 60 years
- Current non-smokers for last 6 months (<5 cigs per week), with a smoking history of <5 pack years
- BMI 20-30 kg/m^2^

**4.2 Exclusion criteria**

- History of allergy
- Upper airway infection in 2 weeks before screening
- Lower respiratory tract infection in past 3 months
- Treatment with local or systemic corticosteroids during previous 2 months
- Signs or symptoms of chronic rhinitis, hypertrophy of turbinates, major septum deviation, nasal polyposis or recurrent sinusitis
- Previous nasal or sinus surgery
- Clinically significant cardiovascular, hepatic, GIT, renal, endocrine, infective, haematological, neurological, dermatological, neoplastic conditions, gastro-oesophageal reflux, depression, TB
- Participation in a therapeutic drug trial in the prior 30 days.
- Medical therapy other than that permitted for contraception.
- Positive pregnancy test
- Inability or unwillingness to use contraception if the patient is a female of child-bearing age.
- History of drug abuse or urine test showing evidence of recreational drug abuse

**5. Lipopolysaccharide (LPS) for Nasal Challenge**

**5.1 LPS from Invivogen**

The highest purity commercially available LPS will be utilised.

We shall employ ultrapure LPS from *Escherichia coli* 0111:B4 (Invivogen Ltd, San Diego, Ca)

This material is authorized for research and laboratory use only, and not for drug use in the USA.

**5.2 Regulatory Aspects**

LPS is given as a challenge agent, to elicit an inflammatory response, and not employed for therapeutic benefit.

Hence the LPS is regarded by the UK regulatory authority (the Medicines and Healthcare Regulatory Authority, MHRA) as a Non-Investigative Medicinal Product (non-IMP) ^(33)^.

For this reason, LPS does not have to be manufactured according to Good Manufacturing Practice (GMP).

The relevant contact person at the MHRA, Clinical Trials Dept is Dr Elaine Godfrey.

**5.3 LPS Stock and Dilution on Day of Use**

5mg of LPS will be diluted in 0.5ml saline to make a single stock of LPS, 0.5ml at 10mg/ml. This dilution will be performed by a physician in the presence of a senior nursing or technical staff, and appropriate documentation will be signed when making up stock as well as when aliquoting and diluting on the day of challenge.

This 10mg/ml stock will be aliquoted as follows:

10 aliquots of 25μl = 250μl total volume

25 aliquots of 10μl= 250μl total volume

There will be storage of all aliquots at -80°C, for a maximum of 1 year, with thawing less than 3h before use.

| **Dilution of stock (10mg/ml)**  **on morning of administration** | **LPS for nasal delivery** | **LPS nasal dose μg per nostril as 100μl actuation** |
| --- | --- | --- |
| 25μl stock  + 225μl saline | 1000μg/ml, vol 250μl | 100 |
| 10μl stock  + 290μl saline | 300μg/ml, vol 300μl | 30 |
| 10μl stock  + 990μl saline | 100μg/ml, vol 1ml | 10 |
| 10μl stock  + 990μl saline  ***Then dilute 10 times***  ***(100μl of diluted stock + 900μl of saline)*** | 10μg/ml, vol 1ml | 1 |

**5.4 LPS administration**

A physician shall prepare the LPS solution, load the Pfeiffer Bidose device and complete a record form. The procedure will be checked and signed by a senior member of ICRRU staff.

LPS solutions (Ultrapure LPS E-Coli 0111:B4 (*Invivogen*) and placebo will be administered from a Pfeiffer Bidose nasal delivery device by a physician.

This is a high quality non-GMP source of LPS.

LPS solution (250μl) will be prepared at 10, 100, 300 and 1000μg/ml,

100μl is administered to each nostril, both nostrils will be sprayed

The dose per nostril corresponds to 1, 10, 30 and 100μg

Weights will be recorded before and after each actuation from the Pfeiffer Bidose.

## Regimen Assignment

Subjects will be assigned to a sequence in accordance with the randomisation schedule generated prior to the start of the study. The randomisation will be balanced over the 5 nasal challenge sequences and will be generated using RandAll, the GSK web-server based clinical trials randomisation validated system.

A description of each challenge sequence is provided in Table 1. The LPS dose corresponds to the dose administered to each nostril.

Table 1. Nasal LPS Dosage Regimen Sequence

| Sequence | Number of subjects receiving sequence | Nasal Challenge 1 | Nasal Challenge 2 | Nasal Challenge 3 | Nasal Challenge 4 | Nasal Challenge 5 |
| --- | --- | --- | --- | --- | --- | --- |
| 1 | 3 | placebo | 1μg LPS | 10μg LPS | 30μg LPS | 100μg LPS |
| 2 | 3 | 1μg LPS | 30μg LPS | placebo | 100μg LPS | 10μg LPS |
| 3 | 3 | 10μg LPS | 100μg LPS | 30μg LPS | 1μg LPS | placebo |
| 4 | 3 | 30μg LPS | 10μg LPS | 100μg LPS | placebo | 1μg LPS |
| 5 | 3 | 100μg LPS | Placebo | 1μg LPS | 10μg LPS | 30μg LPS |

A washout time of 12-28 days will be employed between challenges

1. **Samples and Measures**

**7.1 Samples**

- Nasosorption (right nostril) will be performed before and then 30min, 1h, 2h and hourly to 10h, 24h
- Nasal lavage (left nostril) will be performed at times before (-30 min, 2, 4, 8, 24h.
- Nasal scrape (left nostril) will be carried out after lavage on 4 occasions

……………before (-30 min), 3h, 6h, 24h.

The scrapes will be performed progressively more deeply into the posterior aspect of the nostril, from the inferior aspect of the inferior turbinate

**7.2 Measures**

- Modified TNSS: pain/itching/discomfort, obstruction, sneezing, nasal discharge
- Systemic adverse effects:

fever (record temperature at pre, 2h, 4h, 8h)

flu-like illness (grade severity, 1-3)

myalgia (grade severity, 1-3)

- Nasal sniff pressures, PNIF
- Nasosorption: MSD multiplex (MIP-1α, IL-1β, IL-6, IL-8, TNF-α. TGF-β, IFN-α) from MSD, with analysis at GSK
- Nasosorption for s-ICAM-1 by ELISA, with analysis at GSK
- Nasal lavage cell count and cytospin for cell differential (neutrophils, macrophages, eosinophils, epithelial cells), with assessment at ICRRU
- Nasal scrapes (Rhinoprobe) for gene expression with analysis at GSK

# Data Analysis and Statistical Considerations

## 8.1 Hypotheses

The primary objective for this study is to explore the effects of LPS challenge on ICAM-1 expression in healthy volunteers and determine a dose response relationship. This study is hypothesis generating rather than formal hypothesis testing.

## 8.2 Study Design Considerations

### 8.2.1 Sample Size Assumptions

The study will recruit 15 healthy volunteers in order to ensure 12 subjects complete the study. Any replacement subjects will be assigned to the same regimen sequence as the subject that they are replacing.

No statistical techniques will be used to calculate the sample size. The sample will be based primarily on feasibility.

## 8.3 Data Analysis Considerations

### 8.3.1 Analysis Populations

The ‘All Subjects’ population will be defined as all subjects randomised to nasal challenge sequence who had at least one nasal challenge. This population will be used for all listings, tables and figures.

### 8.3.2 Statistical Analyses

Each of the measures will be summarised descriptively by dose and time using appropriate summary statistics. If appropriate, response variables will be transformed (e.g. log-transformed). Gene expression data will be normalised by house-keeper gene. The TNSS will be calculated by summing the scores of nasal blockage, nasal discharge/rhinorrhoea, nasal burning/itching/pain sensation, and sneezing at each timepoint. Weighted means of symptom scores will be calculated over the time interval 0 to 10h.

TNSS, nasal sniff pressures, nasosorption measures and gene expression data from the nasal scrapes will each be plotted against time, with a separate plot for each subject and measure, and with LPS dose and placebo represented by separate lines on the plots. Summary statistics (e.g. mean +/- 95% confidence intervals or median and interquartile range) for nasal lavage and gene expression data will be plotted by dose against time.

Correlations will be explored using scatter plots for the following measures:

- Weighted means TNSS vs. maximum TNF-α
- Weighted means TNSS vs. maximum IL-8
- Weighted means TNSS vs. maximum s-ICAM-1
- Maximum ICAM-1 expression vs. maximum TNF-α

Reproducibility of the nasal LPS challenge for ICAM-1 expression and TNF-α will be explored using the following subsets of data:

- Predose data for each period to explore intra-subject variability.
  Parameter vs. period number will be plotted with a separate line for each subject to explore whether there is a period effect. Box and whisker plots will also be used to summarise the parameter by period.
- Placebo period data – Parameter vs time will be plotted with a separate line for each subject to explore whether there is a time-course effect. Box and whisker plots will also be used to summarise the parameter over time.

Box and whisker plots for ICAM-1 expression and TNF-α and dose and time will be presented to investigate whether these measures return to baseline after 24h.

Additional exploratory analyses may be performed to further characterize the expression profile of ICAM following nasal LPS challenge.

1. **Study Schedule**

|  | Screening | Sessions 1-5 | | | | | | | | | | | | | | Tel F/up |
| --- | --- | --- | --- | --- | --- | --- | --- | --- | --- | --- | --- | --- | --- | --- | --- | --- |
|  |  | -30m to  -15m | 0 | 30m | 1h | 2h | 3h | 4h | 5h | 6h | 7h | 8h | 9h | 10h | 24h |  |
| Medical interview and examination | X |  |  |  |  |  |  |  |  |  |  |  |  |  |  |  |
| Informed consent | X |  |  |  |  |  |  |  |  |  |  |  |  |  |  |  |
| Height & weight | X |  |  |  |  |  |  |  |  |  |  |  |  |  |  |  |
| BP & heart rate | X | X |  |  |  |  |  |  |  |  |  |  |  |  |  |  |
| Temperature – oral | X | X |  |  |  | X |  | X |  |  |  | X |  |  |  |  |
| Blood sample:  Haematology  Biochemistry | X |  |  |  |  |  |  |  |  |  |  |  |  |  |  |  |
| Pregnancy test | X | X |  |  |  |  |  |  |  |  |  |  |  |  |  |  |
| Drugs of abuse | X |  |  |  |  |  |  |  |  |  |  |  |  |  |  |  |
| ECG | X |  |  |  |  |  |  |  |  |  |  |  |  |  |  |  |
| Nasal lavage to both nostrils and discard (wait 10 mins before sampling) |  | X |  |  |  |  |  |  |  |  |  |  |  |  |  |  |
| Dosing LPS/placebo |  |  | X |  |  |  |  |  |  |  |  |  |  |  |  |  |
| Nasosorption  (Right Nostril) |  | X |  | X | X | X | X | X | X | X | X | X | X | X | X |  |
| Nasal Lavage  (Left Nostril) |  | X |  |  |  | X |  | X |  |  |  | X |  |  | X |  |
| Nasal scrape  (Left Nostril) |  | X |  |  |  |  | X |  |  | X |  |  |  |  | X |  |
| Modified total nasal symptom score, TNSS | X | X |  | X | X | X | X | X | X | X | X | X | X | X | X |  |
| PNIF | X | X |  |  |  | X |  | X |  |  |  | X |  |  | X |  |
| Systemic adverse events: fever, flu, mylagia |  | X |  | X | X | X |  | X |  | X |  | X |  |  | X | X |
